# Case Report: Rescue “awake” extracorporeal membrane oxygenation for acute respiratory failure in severe granulomatosis with polyangiitis with multisystem involvement

**DOI:** 10.3389/fmed.2025.1461269

**Published:** 2025-07-09

**Authors:** Taehun Kim, Byung Wook Song, Hyeong Chan Shin

**Affiliations:** ^1^Division of Pulmonary Medicine, Department of Internal Medicine, Keimyung University School of Medicine, Dongsan Hospital, Daegu, Republic of Korea; ^2^Division of Rheumatology, Department of Internal Medicine, Keimyung University School of Medicine, Dongsan Hospital, Daegu, Republic of Korea; ^3^Department of Pathology, Keimyung University School of Medicine, Dongsan Hospital, Daegu, Republic of Korea

**Keywords:** granulomatosis with polyangiitis, extracorporeal membrane oxygenation, rituximab, acute respiratory distress syndrome, splenic artery vasculopathy

## Abstract

We present the case of a 40-year-old man who developed severe acute respiratory failure along with hemoptysis and was subsequently diagnosed with granulomatosis with polyangiitis (GPA). He was initially treated with high-dose corticosteroids, cyclophosphamide, plasmapheresis, and mechanical ventilation (MV). The patient’s condition deteriorated after being weaned from MV, leading to his transfer to our medical center without reintubation. Upon admission, a high-flow nasal cannula delivering FiO2 of 1.0 was immediately initiated. Despite the severity of hypoxemia, the patient exhibited neither tachypnea nor subjective dyspnea, and was subsequently initiated on “awake” venovenous extracorporeal membrane oxygenation (VV-ECMO) without MV. Anticoagulation therapy was initiated, and continuous renal replacement therapy was commenced to manage anuria associated with acute renal failure. Due to treatment failure after initial immunosuppressive therapy with cyclophosphamide, rituximab was administered as an induction agent. Following four cycles of rituximab, the patient’s respiratory function showed marked improvement. Subsequently, a splenic artery hemorrhage occurred but was effectively managed through prompt embolization, resulting in immediate hemodynamic stabilization. The patient was successfully weaned off VV-ECMO support on day 22 after starting ECMO. After the transfer from the intensive care unit, the patient began active rehabilitation, during which he reported episodes of dizziness. Magnetic resonance imaging of the brain revealed multiple acute infarctions, which are presumed to be caused by vasculitis, leading to the initiation of adjunctive antiplatelet therapy. This represents the first reported case of refractory severe GPA affecting the kidneys, splenic artery, and central nervous system and resulting in respiratory failure, which was managed using “awake” VV-ECMO. The patient remains on maintenance hemodialysis and continues treatment with corticosteroids and rituximab. No disease relapse has occurred until now (June 2025), and the patient is undergoing rehabilitation for intensive care unit-acquired weakness.

## Introduction

Antineutrophil cytoplasmic antibody (ANCA)-associated vasculitis, comprising granulomatosis with polyangiitis (GPA), microscopic polyangiitis, and eosinophilic granulomatosis with polyangiitis, is a systemic disorder that affects small- and medium-sized vessels. Severe cases are characterized by life-threatening multiorgan failure, involving alveolar hemorrhage, glomerulonephritis, central nervous system vasculitis, and cardiac manifestations. Refractory disease is defined as persistent disease activity despite an adequate course of immunosuppressive therapy (1). In GPA, some patients develop acute respiratory distress syndrome (ARDS) secondary to diffuse alveolar hemorrhage. ARDS commonly leads to severe impairment of oxygenation, and its severity is classified based on the ratio of partial pressure of oxygen (pO2) to the fraction of inspired oxygen (FiO2), according to the Berlin Criteria (2). A recent review indicated that although diffuse alveolar hemorrhage is relatively uncommon, it predominantly affects men and critically ill patients, often involves renal impairment, and frequently necessitates MV (3). Current guidelines for severe ARDS recommend the use of rescue extracorporeal membrane oxygenation (ECMO) to decrease mortality rates and reduce ventilator-associated lung injury (4). While venovenous (VV) ECMO is typically employed as a rescue therapy following mechanical ventilation, an alternative approach called “awake” ECMO has been introduced for use in non-intubated patients (5). ECMO has demonstrated success as a rescue therapy in cases of ANCA-associated vasculitis (6), GPA presenting with respiratory failure in adults (7–10), and in pediatric patients (11). This report describes a case of refractory severe GPA that affected the kidneys, splenic arteries, and central nervous system and was complicated by diffuse alveolar hemorrhage, respiratory failure, and cytomegalovirus pneumonitis. The patient was successfully treated using “awake” venovenous ECMO (VV-ECMO) without intubation, alongside induction therapy with rituximab.

### Case description

**Figure 1:**
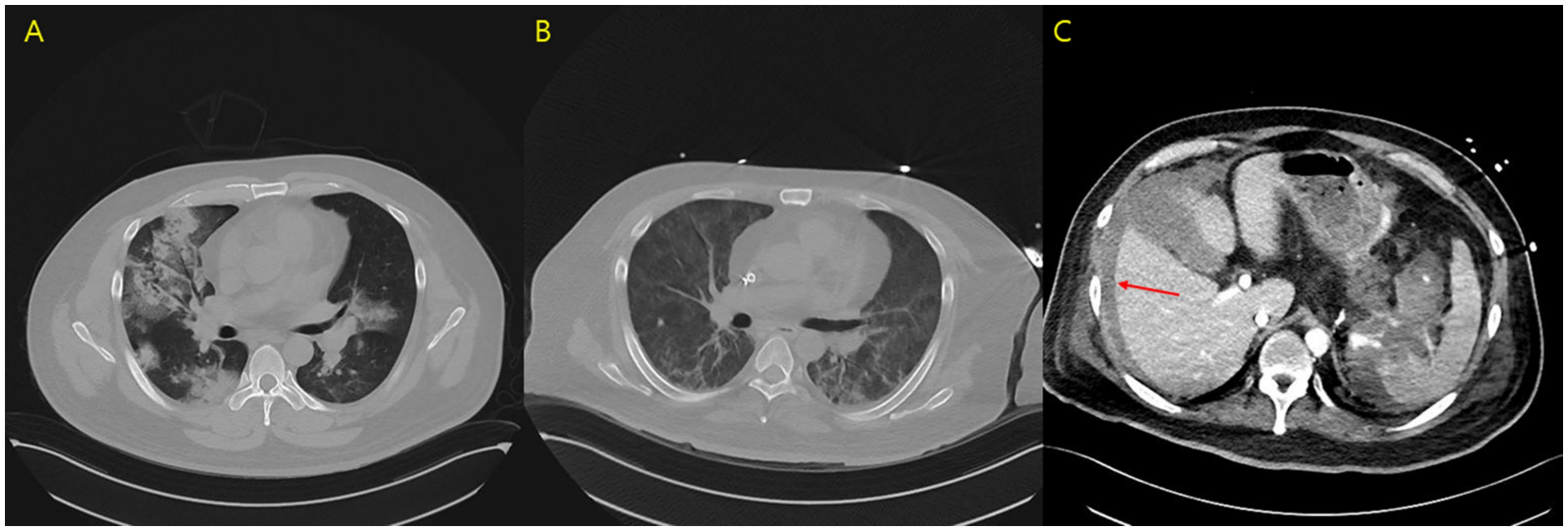
**(A)** Initial chest computed tomography scan. **(B)** Chest computed tomography scan on day 13 of hospitalization. **(C)** Abdominal computed tomography scan on day 13, revealing free fluid collection, hemoperitoneum (arrow), and contrast extravasation from the splenic artery.

A 40-year-old man was admitted to a local medical center’s intensive care unit (ICU) due to respiratory failure and hemoptysis, and MV was initiated immediately (Figure 1A). The patient had no prior medical history and no known family history of autoimmune disease. The patient had a body mass index of 31.9, which is classified as obesity (height: 177 cm; body weight: 100 kg), and his anti-proteinase-3 (PR3) antibody level was greater than 100 U/mL. Progressive renal dysfunction was noted (blood urea nitrogen: 65 mg/dL, serum creatinine: 6.76 mg/dL), and continuous renal replacement therapy was initiated. Induction therapy was initiated with pulse steroids, specifically methylprednisolone, administered at 500 mg every 12 h from 31 August 2023 to 4 September 2023, and cyclophosphamide, administered at 500 mg/m^2^ on 1 September 2023, in response to the anti-PR3 antibody level exceeding 100 U/mL. A diagnosis of diffuse alveolar hemorrhage associated with vasculitis and severe GPA was established. Plasmapheresis was performed on 1 August 2023. After 5 days of pulse steroid therapy, the patient showed clinical improvement and was successfully weaned off MV. The corticosteroid dose was reduced to 125 mg every 12 h from 5 August 2023 to 12 August 2023. However, 7 days later, the patient experienced deterioration in oxygenation, accompanied by recurrent hemoptysis. Consequently, the patient was transferred to our tertiary medical center for advanced respiratory support and rescue therapy.

Upon arrival at the emergency department, the patient was not intubated; therefore, high-flow nasal cannula (HFNC) therapy with FiO₂ of 1.0 was initiated. On presentation, the vital signs upon presentation were as follows: blood pressure 170/68 mmHg, heart rate 114 bpm, respiratory rate 14 breaths per min, and body temperature 36.7°C. Arterial blood gas analysis revealed a pCO₂ of 32.2 mmHg, pH of 7.551, and pO₂ of 67.4 mmHg. Although the pO2/FiO2 ratio was measured without MV and positive end-expiratory pressure, the values met the criteria for severe ARDS. The patient was admitted to the ICU for further respiratory support, and the initiation of rescue therapy was considered. The patient exhibited no signs of respiratory distress. Point-of-care cardiac echo ultrasonography revealed preserved systolic function without significant abnormalities. VV-ECMO was initiated via right internal jugular and left femoral vein cannulation without prior MV. Following ECMO application, the patient’s respiratory pattern remained stable, with subsequent improvement in oxygenation (Table 1). Formal echocardiography conducted after ECMO initiation revealed an ejection fraction of 45%, without evidence of valvular heart disease or pulmonary hypertension. The patient did not report chest pain, and cardiac enzyme levels were within the normal range [troponin I < 0.16 ng/mL (0.0–0.3), CK-MB 1.74 ng/mL (0.0–4.87)]. The patient remained anuric, with renal failure evidenced by a blood urea nitrogen level of 65 mg/dL and a creatinine level of 6.76 mg/dL; therefore, continuous renal replacement therapy was continued via the ECMO circuit. Argatroban was administered for anticoagulation during the ECMO procedure. At the time of ECMO initiation, the patient exhibited thrombocytopenia without the clinical features of disseminated intravascular coagulation. Laboratory findings included a white blood cell count of 6.65 × 10^3^/μL (4.00–10.00), a hemoglobin count of 6.70 g/dL (13.0–17.0), a platelet count of 89 × 10^3^/μL (130–400), a prothrombin time of 13.0 s (10.0–14.0), an activated partial thromboplastin time of 28.8 s (20.0–33.5), and a C-reactive protein level of 6.1 mg/dL (0.0–0.5). Heparin-induced thrombocytopenia could not be ruled out.

**Table 1:** Extracorporeal membrane oxygenation flow sheet.

Day	Pump RPM	Blood flow (L/min)	Air flow (L/min)	FiO_2_ (%)	pH	pCO_2_	pO_2_
D1 (initial)					7.55	32.2	67.4
D1 ECMO	3,510	3.3	2.5	1	7.52	32.6	490
D2	3,510	4.8	2.5	1	7.37	39.9	535
D3	3,510	4.8	2.5	1	7.379	39	512
D4	2,385	4.8	2.5	1	7.39	36.6	391
D5	2,385	3	2	0.95	7.38	33.9	446
D6	2,385	2.9	2.5	0.95	7.44	32.5	390
D7	2,385	2.8	2.5	0.9	7.41	35.5	518
D8	2,385	2.8	2.5	0.9	7.38	34.8	501
D9	2,385	2.8	2.5	0.9	7.4	35	480
D10	2,385	2.8	2.5	0.9	7.42	33.4	445
D11	2,385	2.8	2.5	0.9	7.41	36.7	501
D12	2,385	2.8	2.5	0.85	7.4	35.5	459
D13	2,385	2.9	2.5	0.8	7.42	34.5	461
D14	2,900	2.9	2.5	0.8	7.4	35.2	440
D15	2,700	2.7	2.5	0.75	7.44	33.4	384
D16	2,700	2.7	2.5	0.75	7.4	36.1	390
D17	2,500	2.5	2.3	0.7	7.39	33.4	398
D18	2,500	2.7	2.3	0.7	7.4	33.6	400
D19	2,700	2.5	2.3	0.65	7.28	28.6	400
D20	1,500	1.5	1.7	0.6	7.41	38.4	299
D21	1,650	1.65	1.7	0.5	7.43	34	239
D22	1,600	1.6	0	0	7.37	39.2	102

ECMO, extracorporeal membrane oxygenation; RPM, rotations per minute; FiO_2_, fraction of inspired oxygen; pCO_2_ partial pressure of carbon dioxide; pO_2_, partial pressure of oxygen.

Connective tissue disease-related serological tests were repeated upon admission to our medical center. The results showed no detectable anti-nuclear or anti-double-stranded DNA antibodies. Additionally, antibodies including anti-centromere (0.7 U/mL), anti-glomerular basement membrane (1.4 U/mL), anti-Jo-1 (0.8 U/mL), anti-myeloperoxidase (1.0 U/mL), anti-ribonucleoprotein (1.7 U/mL), anti-Scl-70 (2.1 U/mL), anti-Smith (0.2 U/mL), anti-Ro (0.3 U/mL), and anti-La (2.0 U/mL) were all within the negative range. In contrast, the anti-PR3 antibody remained positive at >100.0 U/mL (Table 2). The patient was diagnosed with refractory severe GPA based on a total score of 8, according to the 2022 American College of Rheumatology/European League Against Rheumatism classification criteria at the time of ICU admission. High-dose corticosteroids combined with rituximab were selected for induction therapy (12). As the patient experienced treatment failure with cyclophosphamide and plasmapheresis, we decided to use rituximab along with pulse steroid therapy. Immunosuppressive therapy for vasculitis was administered concurrently with the initiation of VV-ECMO. High-dose methylprednisolone (500 mg every 12 h) was administered from 15 to 18 August 2023, followed by a tapering regimen: 500 mg/day from 19 to 22 August, 250 mg/day from 23 to 25 August, 125 mg/day from 26 to 28 August, 60 mg/day from 29 August to 2 October, and 30 mg/day beginning 3 October 2023. Rituximab was administered at a dosage of 375 mg/m^2^ weekly, with doses given on 15, 22, and 29 August, followed by a final dose on 6 October 2023. On the day of ICU admission (15 August 2023), cytomegalovirus (CMV) polymerase chain reaction (PCR) testing revealed a viral load of 5,700 copies/mL. However, the results of the CMV PCR became available 1 week later due to processing time. Despite clinical improvement following rituximab induction, qualitative CMV testing of the bronchoalveolar lavage fluid was positive, indicating a possible concurrent CMV infection. Therefore, ganciclovir (250 mg/day; 2.5 mg/kg/day) was administered for 3 weeks. Given the severity of the clinical condition and the patient’s prior hospitalization, additional antimicrobial agents were administered, including carbapenem, teicoplanin, and prophylactic trimethoprim-sulfamethoxazole.

**Table 2:** Autoimmune and laboratory tests flow sheet.

	September 15, 2023	September 22, 2023	September 29, 2023	October 06, 2023	October 31, 2023
Anti-GBM Ab (U/mL)	Negative (1.4)	Negative (0.3)	Negative (0.5)	Negative (0.9)	Negative (0.1)
Anti-MPO Ab (U/mL)	Negative (1.0)	Negative (0.8)	Negative (0.8)	Negative (0.8)	Negative (0.7)
Anti-PR3 Ab (U/mL)	Positive (>100)	Positive (27.1)	Positive (13.3)	Positive (5.4)	Positive (6.6)
CMV DNA Real-time PCR (copies/mL)	5,700	14,356	24,746	74,569	15,365
KL-6 (U/mL)	562.1	693.6	972.8	631.8	
Interleukin 6 (pg/mL)	30.4				

Anti-GBM Ab (negative <20 U/mL), anti-glomerular basement membrane antibody; anti-MPO Ab (negative <5 U/mL), anti-myeloperoxidase antibody; anti-PR3 Ab (negative <5 U/mL), anti-proteinase-3 antibody; CMV DNA, cytomegalovirus deoxyribonucleic acid; KL-6, Krebs von den Lungen-6; PCR, polymerase chain reaction.

**Figure 2:**
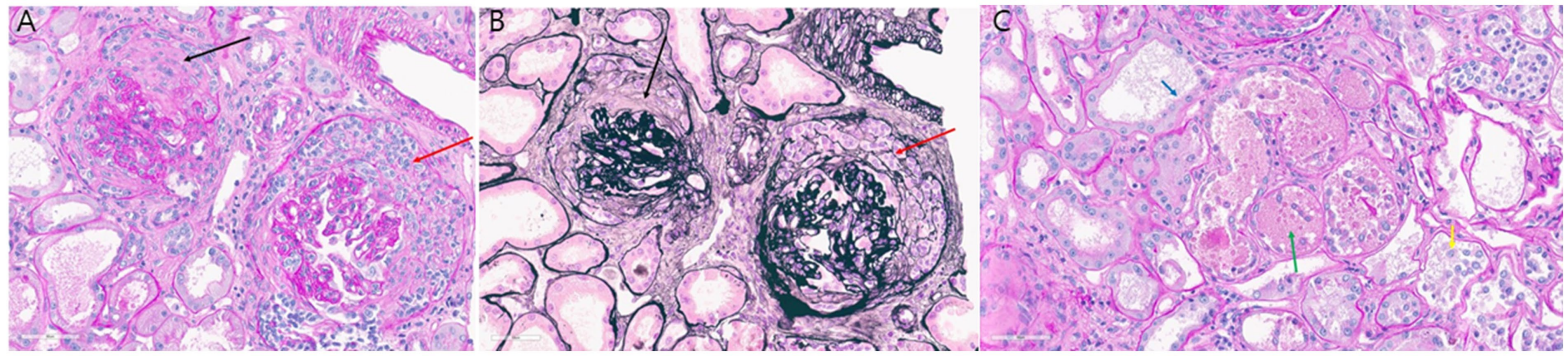
**(A,B)** Glomerular crescents: **(A)** circumferential fibrocellular crescent (black arrow) and cellular crescent (red arrow) accompanied by glomerular basement membrane wrinkling (periodic acid–Schiff stain; original magnification, ×40); **(B)** corresponding findings stained with periodic acid–methenamine silver (original magnification, ×40). **(C)** Acute tubular necrosis: loss of brush border (blue arrow), denuded tubular epithelial cells (yellow arrow), and tubular cast containing fibrin debris (green arrow) as seen on periodic acid–Schiff staining (original magnification, x40).

Anticoagulation therapy with argatroban was administered during the treatment period, and no further episodes of hemoptysis were observed. Following medical management, including volume reduction via continuous renal replacement therapy, chest radiography showed improvement in bilateral parenchymal consolidations. The anti-PR3 antibody level decreased from >100 U/mL on day 1 to 27.1 U/mL on day 8 (Table 2), and oxygenation parameters showed progressive improvement (Table 1; Figure 1B). On day 13 following ECMO initiation, although hemoptysis did not recur, a sudden drop in hemoglobin levels was detected along with hypotension. The patient reported dull abdominal pain, and point-of-care ultrasonography revealed the presence of fluid in the right subphrenic area. Consequently, chest and abdominal computed tomography scans were performed, revealing hemoperitoneum caused by splenic artery bleeding with contrast dye extravasation, which was presumed to be secondary to underlying vasculopathy (Figure 1C). A blood transfusion was conducted, consisting of four units of packed red blood cells (400 mL) and two units of fresh frozen plasma (400 mL), and anticoagulation therapy was discontinued. Emergent angioembolization of the splenic artery was promptly performed (Supplementary Figures S1A,B). No further decline in hemoglobin levels was observed after the embolization, and the patient’s hemodynamic status stabilized rapidly, with normalized blood pressure and heart rate, leading to the discontinuation of nootropics. By day 21, the FiO2 of HFNC was reduced to 0.6. As the patient remained clinically stable, ECMO weaning was initiated. The patient was successfully weaned from VV-ECMO support on day 22 after starting ECMO. The patient exhibited favorable clinical recovery, enabling a gradual reduction and eventual discontinuation of supplemental oxygen. A renal biopsy was performed to confirm the diagnosis, which demonstrated crescentic glomerulonephritis (Figures 2A,B) and acute tubular necrosis (Figure 2C). As the anuric state persisted and renal biopsy findings suggested irreversible damage, long-term hemodialysis was initiated via a permanent catheter. During active rehabilitation, the patient experienced episodes of dizziness. Magnetic resonance imaging of the brain revealed multiple acute infarctions in the superior right frontal cortex and left parietal cortex (Supplementary Figures S2A–C). The cerebral infarctions were attributed to vasculitis; therefore, an additional antiplatelet agent, aspirin 100 mg/day, was prescribed. After receiving intensive care, the patient was discharged with ongoing maintenance therapy including hemodialysis, corticosteroids, and rituximab. No relapse has been observed until now (June 2025), and the patient continues rehabilitation for ICU-acquired weakness.

## Discussion

A case of severe GPA was presented, in which induction therapy with cyclophosphamide was unsuccessful; however, successful disease control was achieved following rituximab-based induction (Supplementary Figure S3). As clinical improvement was consistently observed following rituximab induction, the deterioration before hospitalization was attributed to cyclophosphamide induction failure rather than CMV pneumonia. Given the patient’s critical condition and the requirement for ongoing immunosuppressive therapy, ganciclovir was concurrently administered. In the critical care setting, this case represented successful management of severe ARDS using ECMO without MV as a rescue therapy. The patient initially presented with multiorgan dysfunction involving both the pulmonary and renal systems. Additionally, the involvement of both the splenic artery and the cerebrovascular system was identified. Splenic artery involvement is uncommon and has been documented in a limited number of cases (13–16). Cerebrovascular events are also infrequent; although central nervous system involvement was reported in 32.3–33.6% of patients, cerebrovascular manifestations occurred in only 4.0% of 146 patients (17, 18).

For the rescue management of ARDS, both VV-ECMO and prone positioning are considered viable options. Prone positioning is strongly recommended in conjunction with MV (19). However, recent evidence indicates that prone positioning does not significantly reduce the duration of successful weaning or mortality when compared to the supine position in patients undergoing VV-ECMO (20). In this case, the clinical team had to decide between initiating invasive mechanical ventilation with prone positioning or proceeding with VV-ECMO as the initial rescue therapy. Initial concerns were raised regarding the use of prone positioning; however, due to the absence of labored breathing, VV-ECMO with induction therapy was initiated without mechanical ventilation, and prone positioning was consequently not implemented. Anticoagulation was maintained during VV-ECMO, which may have contributed to the splenic artery hemorrhage in the context of underlying vasculopathy. Therefore, the use of anticoagulants during ECMO should be cautiously considered in severe GPA cases with multiorgan involvement. ARDS is a frequent complication of severe GPA and often necessitates ECMO support (9). In selected cases, “awake” ECMO without endotracheal intubation is feasible; however, meticulous monitoring of bleeding complications caused by vasculopathy is essential.

During the SARS-CoV-2 pandemic, it is essential to carefully assess the etiology of disorientation in patients with respiratory failure, particularly in immunocompromised individuals receiving immunomodulatory therapies. GPA associated with ARDS may clinically resemble infectious diseases that necessitate distinct treatment approaches (21, 22). PCR testing at our center was negative for SARS-CoV-2; however, CMV infection was confirmed. In patients with autoimmune diseases, both infection and the level of immunosuppression must be carefully managed to maintain effective disease control. Although this can be challenging, treatment decisions should be guided by the patient’s clinical trajectory.

Although diagnostic biopsy plays a crucial role, its timing may be deferred based on the patient’s clinical severity and overall condition, as seen in this case. However, when ANCA positivity indicates a severe disease course, such as in GPA, timely initiation of appropriate therapy is essential. Timely induction with an appropriate agent significantly improves survival outcomes. A randomized, double-blind, non-inferiority trial demonstrated that a rituximab-based regimen is not inferior to a cyclophosphamide-based regimen (23–25). For induction therapy in GPA, current options include rituximab or cyclophosphamide, both administered in combination with glucocorticoids (26). However, the 2021 American College of Rheumatology/Vasculitis Foundation guidelines recommend rituximab over cyclophosphamide for induction therapy in severe GPA (1). Successful induction can still be achieved in patients with prior treatment failure or early relapses following immunosuppressive therapy, as demonstrated in this case.

In conclusion, this report describes a case of severe GPA with multiorgan involvement, including pulmonary, renal, splenic arterial, and central nervous system manifestations. The condition was successfully managed using “awake” VV-ECMO in combination with rituximab-based induction therapy. This case underscores the importance of guideline-directed therapy when selecting induction regimens. For carefully selected patients, “awake” VV-ECMO without MV may offer a viable alternative to the routine application of invasive ventilation.

## Data Availability

The datasets presented in this article are not readily available because the data that support the findings of this study are not openly available due to the fact that consent to share data was not obtained from participants. However, the datasets used and analyzed in the current study are available from the corresponding author on reasonable request. Requests to access the datasets should be directed to TK, taehunlung@gmail.com.

## Supplementary material

SUPPLEMENTARY FIGURE S1 Angiographic images obtained before **(A)** and after **(B)** splenic artery embolization. Contrast extravasation.

SUPPLEMENTARY FIGURE S2 Brain magnetic resonance images of superior frontal cortex infarction **(A)** and left parietal cortex infarction **(B,C)**.

SUPPLEMENTARY FIGURE S3 Flow chart.
