# Supplementary figures and images for: Case Report: Rescue “awake” extracorporeal membrane oxygenation for acute respiratory failure in severe granulomatosis with polyangiitis with multisystem involvement
Source: Front Med (Lausanne). 2025 Jul 9;12:1461269. doi: 10.3389/fmed.2025.1461269 (PMC12283735; doi:10.3389/fmed.2025.1461269)

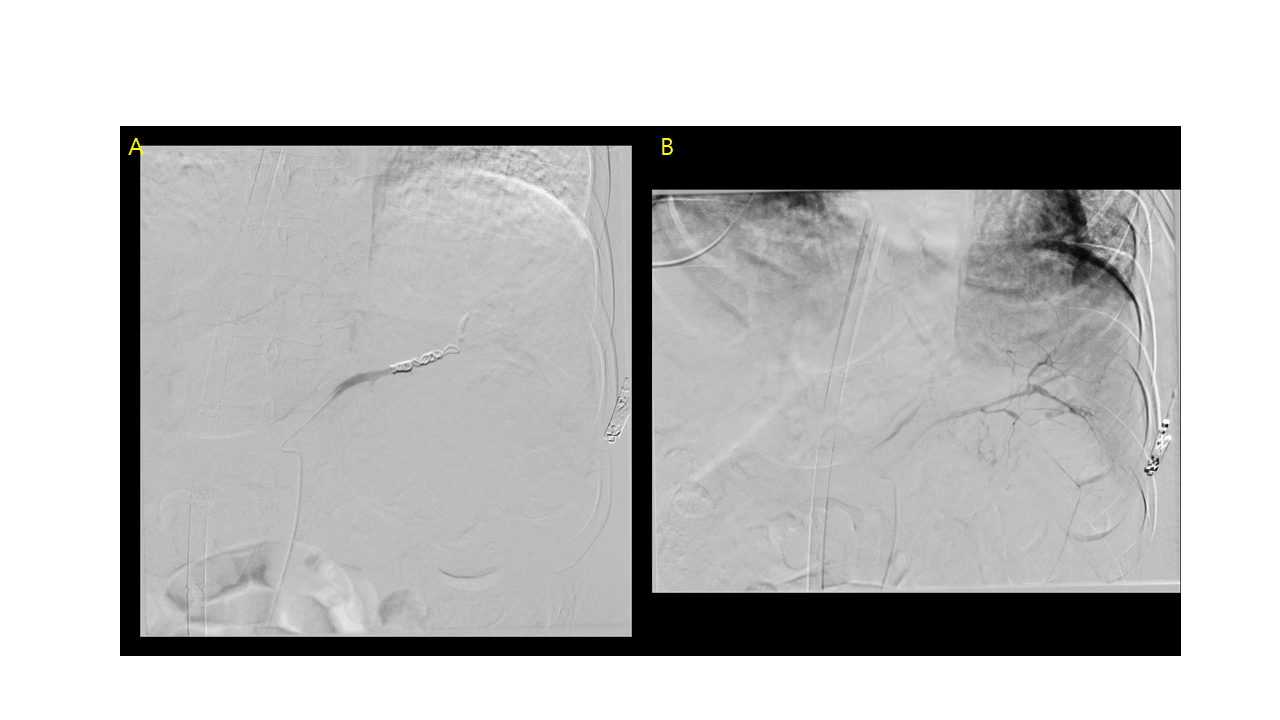

Supplement: SUPPLEMENTARY FIGURE S1 — Angiographic images obtained before (A) and after (B) splenic artery embolization. Contrast extravasation. [file Image_1.tif]

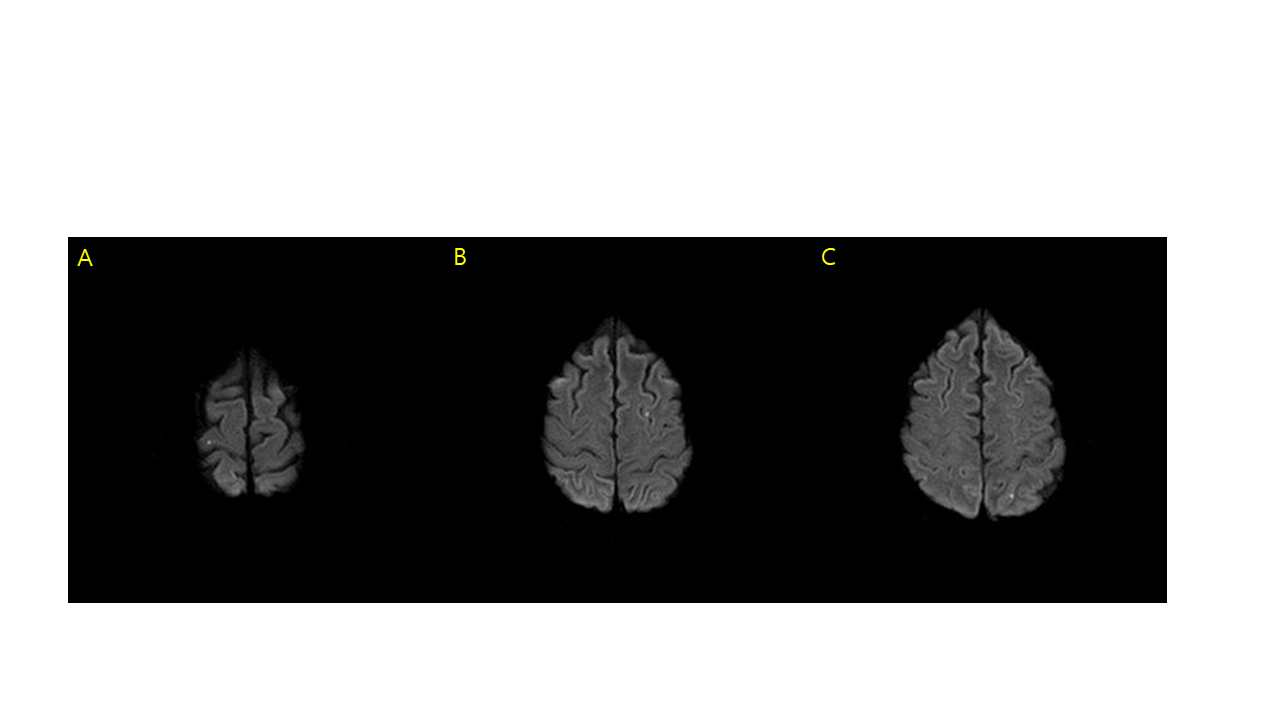

Supplement: SUPPLEMENTARY FIGURE S2 — Brain magnetic resonance images of superior frontal cortex infarction (A) and left parietal cortex infarction (B,C). [file Image_2.tif]

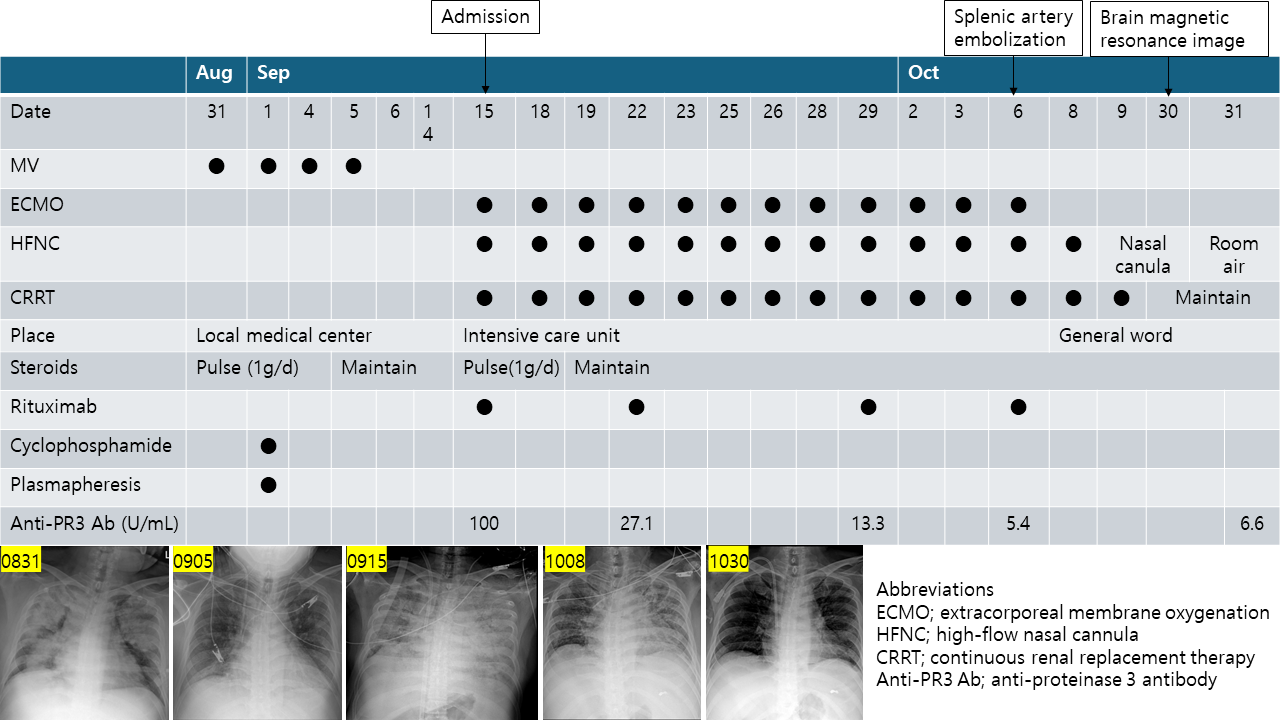

Supplement: SUPPLEMENTARY FIGURE S3 — Flow chart. [file Image_3.tif]
